# Single-Stage Pedicle Preputial Tube Substitution Urethroplasty with Corpora Cavernosa Augmentation Using Buccal Mucosa Graft for Primary Peno-Scrotal Hypospadias Repair in Adults

**DOI:** 10.1590/S1677-5538.IBJU.2024.0650

**Published:** 2025-03-25

**Authors:** Pankaj Joshi, Sanjay Kulkarni, Nicole Albanese, Fausto Negri, Marco Bandini

**Affiliations:** 1 Kulkarni Reconstructive Urology Center Department of Urology Pune India Kulkarni Reconstructive Urology Center, Department of Urology, Pune, India; 2 Urological Research Institute Division of Experimental Oncology/Unit of Urology IRCCS Ospedale San Raffaele Milan Italy Division of Experimental Oncology/Unit of Urology, Urological Research Institute (URI), IRCCS Ospedale San Raffaele, Milan, Italy

## Abstract

**Introduction:**

Pre-engagement hypospadias repairs are not uncommon in developed countries like India. Male genital malformations that are not associated with voiding dysfunction are often underreported by families or male patients until the boy reaches marriageable age. At that point, they seek consultation, fearing rejection by potential partners and desiring a rapid and possibly single-stage repair. Therefore, it is not uncommon for primary repair to be performed after puberty, once the penis has fully developed. This may also have consequences on the complexity of surgical repair (1), given that ventral chordee can alter penile development, leading to a higher degree of corporal fibrosis and consequently a more severe ventral curvature. Additionally, the proportion between penile dimensions and craniofacial dimensions is not constant throughout childhood. Genitals are underdeveloped during prepubescence, while the craniofacial region reaches adult dimensions more rapidly, resulting in tissues like buccal mucosa being more abundant compared to adults for pendular urethra reconstruction. These concepts are crucial when planning primary hypospadias repair in adults, as the severity of genital hypospadias may be greater and graft availability may be insufficient.

**Surgical technique:**

In our practice, it has not been uncommon to encounter cases of primary hypospadias repair where common techniques such as Asopa (2) buccal mucosa graft (BMG) urethoplasty or Bracka two-stage repair were not applicable due to limited availability of BMG to reconstruct the entire penile urethra. In this article, we aim to describe a technique for repairing severe primary hypospadias, where the urethra is reconstructed in a single stage using a pedicle preputial tube, and severe chordee resulting from delayed hypospadias repair combined with corporal fibrosis is resolved through BMG grafting.

Patients are typically assessed preoperatively to evaluate the development of the glans for a glansplasty, the availability of the prepuce, and the condition of the buccal mucosa on both cheeks. Subsequently, surgery is performed under general anesthesia with the patient in a supine position. Initially, artificial erection is induced to accurately gauge the severity of curvature. This technique is typically reserved for severe cases of hypospadias where the ventral curvature exceeds 60°. Degloving is then carried out while preserving the vascular support of the prepuce. A circumferential incision is made 5 mm below the coronal sulcus, and both the skin and the dartos are dissected up to the level of Buck's fascia. It is crucial to preserve the vascularization of the dartos during this step, as failure to do so may prevent subsequent flap harvesting. Next, the curvature is reassessed. If a severity above 60° is confirmed, the urethra is transected. However, this step often does not fully resolve the ventral chordee, as the development of the two corpora bodies may have been compromised by the shortened urethra, leading to frequent ventral fibrosis. To straighten the penis, ventral corporotomies are performed. If necessary, a Nesbit procedure is conducted, involving the removal of a wedge of fibrotic tunica albuginea without suturing the two edges. This approach avoids affecting the final length of the penis through plication of the tunica albuginea. Instead, we opt to patch the albuginea defect with buccal mucosa graft. Once the albuginea graft is secured, the straightness of the penis is reassessed to confirm the resolution of the chordee. Finally, urethral reconstruction is performed. A pedicle preputial flap is harvested following the standard technique (3). Careful attention is given to mobilizing the preputial skin from the underlying dartos. Once harvested, the flap is tubularized over a 14 Ch Foley catheter and anastomosed to the proximal urethral end. Subsequently, glans wings are developed, and the distal end of the preputial tube is positioned over the glans sulcus. Glans wings are then closed in two layers over the tube. Placing the suture line of the tubularized flap towards the corpora bodies may reduce the risk of fistula formation. Finally, the penile skin is closed over and sutured to the glans sulcus. A compressive dressing is maintained for two days. The catheter is left in place for three weeks and then removed without performing pericatheter urethrography.

**Results:**

This is a prospective study done at our Institute from July 2016 to July 2023. A total of 23 male patients were included in the study. Age range was from 16 years to 27 years. All patients underwent correction in a single stage. For 2 patients bovine pericardium was used for corporal augmentation. For remaining 21 patients buccal graft was used for corporal augmentation. All patients had urethral reconstruction in single stage using the pedicled preputial tube. Catheter was kept for 6 weeks. Minimum follow up was 6 months. Two patients developed meatal stenosis which was managed by meatotomy. Two patients developed urethro-cutaneous fistula which was repaired after 6 months. Three patients developed anastomotic narrowing at the proximal junction of pedicled tube and native urethra. They were initially managed with dilatation. They required a small dorsal inlay BMG graft with Asopa technique and are now doing well. One patient with bovine pericardium used developed wound complications and complete dehiscence. He was reoperated at 1 year interval. One patient developed distal penile skin blackening which was treated conservatively. Overall, 20 patients had successful outcome in first surgery. No patient required any reintervention for chordee.

**Conclusions:**

Single-stage pedicle preputial tube substitution urethroplasty with corpora cavernosa augmentation using BMG for primary peno-scrotal hypospadias repair in adults is safe and feasable surgery, despite a 30.4% complication rate. This is the option for penile lengthening as well as single stage urethral reconstruction.

Available at: http://www.intbrazjurol.com.br/video-section/20240650_Negri_et_al
